# Enhanced magnetic second-harmonic generation in an ultra-compact plasmonic nanocavity

**DOI:** 10.1038/s41377-025-01962-3

**Published:** 2025-09-05

**Authors:** Yaorong Wang, Ilya Razdolski, Shixuan Zhao, Fan Yang, Xiu Liang, Yuri Kivshar, Dangyuan Lei

**Affiliations:** 1 Department of Materials Science and Engineering, Centre for Functional Photonics, and Hong Kong Branch of National Precious Metals Material Engineering Research Centre, City University of Hong Kong, Hong Kong, China; 2 Department of Physics, City University of Hong Kong, Hong Kong, China; 3 College of Physics and Key Laboratory of High Energy Density Physics and Technology of the Ministry of Education, Sichuan University, Chengdu, Sichuan China; 4 Advanced Materials Institute, Qilu University of Technology (Shandong Academy of Sciences), Jinan, Shandong China; 5 Nonlinear Physics Centre, Research School of Physics, Australian National University, Canberra, Australia

**Keywords:** Nonlinear optics, Nanophotonics and plasmonics

## Abstract

Observation of the second-harmonic generation (SHG) from subwavelength metallic structures is often hindered by the interrelations of higher-order multipolar contributions. In particular, the magnetic Lorentz contribution to SHG is often neglected due to the ineffective magnetic field enhancement in electrically resonant structures. Here, we demonstrate a strong Lorentz-driven SHG output at the plasmon-induced magnetic dipolar resonance in inversion-symmetry-broken plasmonic nanocavities. We observe experimentally tenfold enhancement in the SHG intensity when the magnetic dipole mode is excited, with polarization-resolved measurements confirming the significant role of the hydrodynamic Lorentz-driven second-order nonlinear response. The enhancement originates from a significant spatial overlap between the electric and magnetic fields within the nanometer-scale cavity gaps. Our findings outline the critical role played by the resonant Lorentz-driven optically induced magnetic nonlinearities in metallic nanocavities, and it paves the way towards developing highly efficient nanoscale nonlinear photonic devices.

## Abstract (web summary)

We demonstrate the Lorentz-driven second-harmonic generation using magnetic dipole resonance in an ultra-compact plasmonic nanocavity. Enhanced electromagnetic fields are confined and spatially overlapped, enabling tenfold enhancement.

## Graphical abstract

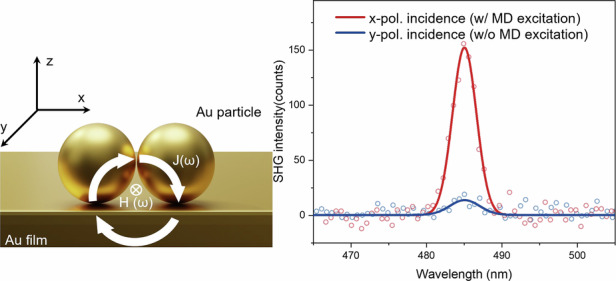

## Introduction

Optically induced magnetic dipole resonances, similar to their electric counterparts, can effectively be engineered in metallic split-ring resonators^1^ or high-index dielectric subwavelength particles^2,3^ through judicious nanostructuring. The emergence of the magnetic dipole modes in subwavelength optical structures introduces a distinct set of physical phenomena enabling their promising functionalities such as field detection^4,5^, Raman spectroscopy^6^, phase engineering^7,8^, Fano resonances^9–11^, nonradiating anapole modes^12^, and enhanced optical nonlinearity^13,14^. Among these applications, the second-harmonic generation (SHG) stands out as a process of significant interest. Over the decades, SHG has been investigated across diverse nanostructures, including double-resonant mode-matching antennas^15^, Fano-resonant nanoclusters^16,17^, nanoparticle lattices^18^, and symmetry-broken nanostructures^19–21^. These studies have revealed promising prospects for the application of SHG in various fields, including sensor^22,23^, biomedicine^24^, optical encryption and data storage^25^, quantum information processing^26^, and the advancement of nonlinear-optical devices^27–29^.

Understanding the physics and origin of nonlinear-optical responses of metallic nanostructures is a long-standing fundamental problem^30^. Soon after the initial observation of SHG, it was realized that in metallic systems with inversion symmetry, the second-order nonlinear-optical response is governed by surface dipole, bulk electric quadrupole, and magnetic dipole contributions. The first phenomenological attempts at qualitative description have later evolved into a free electron model for second-order susceptibility^31^. Later, when its inadequacy was demonstrated in the vicinity of metal surface^32^, a hydrodynamic model (HDM) for the nonlinearity of the electron gas has been developed^33,34^. Despite a few known issues with its quantitative predictions and disagreement with results of the time-dependent local density functional calculations^35^, HDM occupies an important place in modern nonlinear nanophotonics^36–42^.

Arguably, the appealing physical transparency of the HDM plays a pivotal role in elucidating the intricate physical processes that govern SHG. The second-order nonlinear-optical response is broken into three main contributions that originate in the Coulomb interaction, the Lorentz force, and the convective electron dynamics^37^. Within this approach, the convective and Coulomb contributions related to the electric properties are typically regarded as the primary SHG sources in designing nonlinear nanostructures^43,44^. Although the Lorentz-induced SHG was observed in split-ring resonators^44^ and T-shaped apertures^45^, the interplay between Lorentz and convective contributions complicates the unambiguous identification of the SHG enhancement mechanism^46–48^. The ongoing challenges in disentangling and utilizing these nonlinear mechanisms require an improved understanding of the relative SHG contributions and their interrelations in plasmonic nanocavities.

In this work, we present an in-depth analysis of the magnetic Lorentz contribution to SHG in plasmonic dimer-on-film nanocavities. By exciting the magnetic dipole resonance, we demonstrate both experimentally and theoretically a strong Lorentz-driven SHG. Unlike previous studies, we employ a bottom-up synthesis approach to assemble an ultra-compact nanocavity, effectively confining the electromagnetic field. Our approach can be extended to a large class of nanophotonic systems, enabling a dominant magnetic Lorentz force contribution in the ultra-compact nonlinear nanophotonic device.

## Results

### Origins of SHG

The hydrodynamic model provides a framework for describing the polarization inside metals. The nonlinear source due to complex dynamics of the electron gas in the electromagnetic field, is given by ref. ^44^

1 $$\begin{array}{l}{{\bf{S}}}_{{\rm{NL}}}=\frac{e}{{m}_{e}^{* }}{{\bf{E}}}_{1}\left(\nabla \cdot {{\bf{P}}}_{1}\right)\\\qquad +\frac{i\omega \mu e}{{m}_{e}^{* }}{{\bf{P}}}_{1}\times {{\bf{H}}}_{1}\\\qquad-\frac{{\omega }^{2}}{{n}_{0}e}\left(\left(\nabla \cdot {{\bf{P}}}_{1}\right){{\bf{P}}}_{1}+\left({{\bf{P}}}_{1}\cdot \nabla \right){{\bf{P}}}_{1}\right)\end{array}$$

Here, *e* and ${m}_{e}^{* }$ denote the electron charge and mass, *n*_0_ ≈ 6 × 10^22^ cm^−3^ is the electron density in Au, *µ* is the magnetic permeability. The subscript 1 indicates the fields at the fundamental frequency *ω*. The nonlinear source given by Eq. 1 consists of three main contributions^37^. The first contribution proportional to ${{\bf{E}}}_{1}\left(\nabla \cdot {{\bf{P}}}_{1}\right)$ is the Coulomb term, which is nonzero only near the metal surface. The magnetic Lorentz force contribution, expressed as ${{\bf{P}}}_{1}\times {{\bf{H}}}_{1}$, is proportional to the cross-product of the electric and magnetic fields, which is purely a bulk term. The last convective term, $\left(\nabla \cdot {{\bf{P}}}_{1}\right){{\bf{P}}}_{1}+\left({{\bf{P}}}_{1}\cdot \nabla \right){{\bf{P}}}_{1}$, contains surface and bulk contributions. The quantum pressure term in the model is not considered, as its effects are primarily significant for subnanometer gaps^39,43^.

Among these terms, the Lorentz contribution arises from the interaction between the electric and magnetic fields, and it can be significantly amplified by enhancing the magnetic field in specifically designed structures. However, studies of SHG in nanoscale split-ring resonators, known for their magnetic response, indicate that the Lorentz contribution remains negligible^44^. The relative weakness of the Lorentz term originates in its dependence on the cross-product of electric and magnetic fields. In many resonant plasmonic nanostructures, the maxima of these fields do not coincide spatially, which is obtained directly from numerical simulations^49–51^. This spatial misalignment reduces the cross-product ${{\bf{P}}}_{1}\times {{\bf{H}}}_{1}$ required for Lorentz-driven SHG, especially inside the metal. Ultra-compact plasmonic nanocavities with ultrasmall (on the order of 1 nm) gaps can generate stronger confined fields within sub-diffraction-limited mode volumes^52^. To make the Lorentz contribution dominant, both magnetic field enhancement and the overlap between the electric and magnetic fields are essential.

**Fig. 1:**
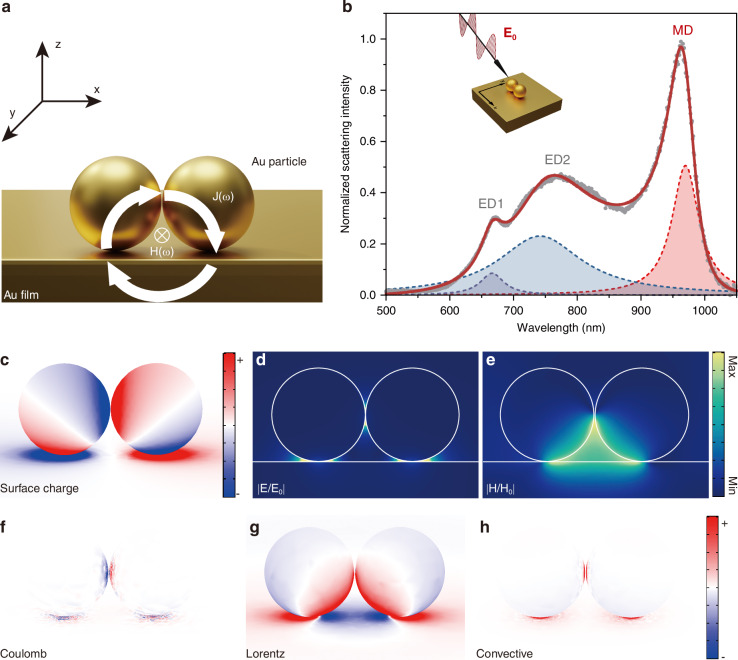
Excitation of the magnetic dipole (MD) mode in a plasmonic dimer-on-film nanocavity. **a** Schematic illustration of the strategy using MD in a dimer-on-film nanocavity to enhance the Lorentz contribution to SHG. **b** Experimentally measured (dots) and fitted (solid line) scattering spectra of the nanocavity. The shaded areas illustrate the contributions from individual resonances. **c** Charge distributions, **d** electric field enhancement maps, and **e** magnetic field enhancement maps of magnetic dipole. **f** Coulomb, **g** Lorentz, and **h** Convective contribution to the second-order charge distributions at 2*ω*

To this end, we study nonlinear-optical properties of a plasmonic nanocavity in the dimer-on-film geometry, which enables the excitation of a magnetic dipole (MD) resonance^53,54^. Pairs of 100 nm diameter gold spheres encapsulated with ~2 nm silica shell are positioned on top of a smooth Au film, forming an ultra-compact plasmonic nanocavity, as depicted in Fig. 1a. For a detailed description of the fabrication process of the dimer-on-film cavity, see Materials and Methods and Supplementary Section 1. The silica forms stable gaps between the nanospheres and the Au film, which serve as capacitances in the ternary LC resonance for exciting MD. Each stable gap is essential for the formation of the MD mode, and once the gap is bridged, it no longer exhibits the characteristics of an MD mode. The light-induced circulating current **J**(*ω*) generates a resonantly enhanced magnetic field **H**(*ω*) in the direction perpendicular to the *xz* plane.

Using our home-built oblique-incidence dark-field scattering microscopy, we precisely define the incident angle and polarization to measure the scattering response of the cavity. Under p-polarized excitation at an incident angle of 70°, a strong peak is observed in the scattering spectrum at *λ* ≈ 950 nm, see Fig. 1b. Following previous work^51^, we attribute this peak to the MD mode. The MD resonance has a linewidth of approximately 50 nm (*Q* ≈ 19), while the two electric dipole modes are also observed, labeled as ED1 (at 660 nm) and ED2 (at 750 nm). At ED1, the induced electric dipole moment in the dimer is parallel to the metal film. ED2 has a pronounced vertical electric dipole moment, featuring opposite-sign surface charges on the particle and the film. To illustrate the nature of the MD resonance, we plot the surface charge distribution in Fig. 1c, revealing the circulating displacement currents induced by the resonant optical excitation. Figure 1d and Fig. 1e show the spatial distributions of the normalized electric *|***E***/***E**_0_*|* and magnetic *|***H***/***H**_0_*|*fields in the mirror symmetry (*xz*) plane of MD mode. The magnetic field hotspot is mainly confined in the triangular region bounded by the nanosphere dimer and the film. Notably, in this hotspot, both the magnetic and electric fields are simultaneously enhanced, with maximum field amplitude enhancement factors exceeding 300 and 25, respectively. Compared to the ED2 mode (Q ≈ 5), MD mode does not exhibit significantly greater local electric field enhancement despite its higher Q-factor (see Supplementary Section 3.3). Instead, the MD mode predominantly enhances the magnetic field, with notable spatial overlap between the enhanced magnetic and electric fields. This spatial overlap is a unique feature of MD resonance compared to these electric dipole modes, and it is crucial for the Lorentz-driven SHG response predicted by HDM.

To further illustrate the nonlinear contributions, Fig. 1f–h presents the spatial distributions of the second-harmonic components, including the Coulomb, Lorentz, and convective second-order surface charge, respectively. These distributions provide direct insights into the individual contributions of each term to the SHG response. In particular, the Lorentz contribution, proportional to the cross-product ${{\bf{P}}}_{1}\times {{\bf{H}}}_{1}$, exhibits a pronounced enhancement within the region of significant spatial overlap between the electric and magnetic fields. Enabled by the ultra-compact dimer-on-film geometry, this pronounced cross-product is further corroborated by quantitative analysis presented later, underscoring its pivotal role.

### Enhanced magnetic SHG

**Fig. 2:**
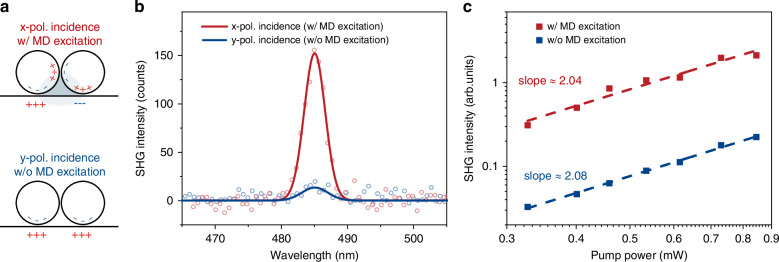
Comparison of SHG intensity with and without MD excitation. **a** Schematic representation of two distinct modes under orthogonal incident polarizations: x-polarized (red) and y-polarized (blue). **b** SHG spectra of the dimer-on-film cavity for the two orthogonal polarization incidences. **c** Power dependence of SHG intensity for the two polarization incidences

To experimentally validate the SHG enhancement by the MD mode, we conducted spectroscopic nonlinear-optical measurements in the vicinity of the MD resonant wavelength *λ*_MD_ ≈ 950 nm. A pulsed femtosecond laser beam with a Gaussian profile was employed as the excitation source for SHG. Given that the electric field component along the dimer axis (x-direction) is crucial for the excitation of the MD, a half-wave plate (HWP) was introduced in the incident optical path to manipulate the polarization, thereby enabling selective excitation or suppression of the MD mode. When the incident polarization is parallel to the x-direction, the SHG signal reaches its maximum as the fundamental wavelength is slightly redshifted from *λ*_MD_ to ~970 nm. When the HWP was rotated to align the incident polarization along the y-direction, a substantial decrease in SHG intensity was observed. In our experiments, the excitation laser beam was tightly focused using a high-numerical-aperture (NA = 0.95) objective lens. This high NA inherently introduces a broad range of incident angles and substantial out-of-plane (z direction) electric field components. According to vectorial diffraction theory (see Supplementary Section 3.2 for further details), the out-of-plane electric field component **E**_z_ contributes significantly to the total field intensity at the focal spot (~17%). Under x-polarized incidence, the in-plane **E**_x_ dominates the Lorentz-driven SHG. Notably, the MD resonance frequency remains independent of the incident angle (see Fig. S3b), indicating that the SHG enhancement at the MD resonance can be consistently attributed to the Lorentz term in various excitation configurations. Under y-polarized incidence, the SHG response is primarily attributed to the vertical electric dipole component oriented perpendicular to the gold film, as illustrated in Fig. 2a. Figure 2b presents a comparative analysis of the SHG intensity at 970 nm for x-polarized excitation, where the MD mode is excited (red), and y-polarized excitation, where the MD mode is suppressed (blue). The SHG intensity for the x polarization is approximately ten times that for the y polarization. By fitting the recorded spectrum with a Gaussian profile and integrating it, we can obtain the SHG intensity. As shown in Fig. 2c, measured SHG intensity increases quadratically with the average power of the fundamental radiation in the used experimental range (0.3–0.9 mW), which not only reflects the intrinsic nonlinear nature of SHG but also indicates that no structural modification occurred in the samples during the measurements. High-power femtosecond laser excitation may induce thermal effects, such as annealing, melting, or ablation, leading to structural changes in plasmonic nanocavities. Such effects can result in a superquadratic SHG slope^55,56^. While thermal effects under intense excitation are often analyzed using the two-temperature model (TTM)^57^, such transient dynamics are beyond the scope of this study, which focuses on steady-state SHG signals averaged over many pulses. To quantitatively evaluate the nonlinearity, we define the SHG conversion efficiency as *η* = *W*_2_*/W*_1_^2^, where W_2_ and W_1_ refer to the average power of SHG and fundamental radiation, respectively. Our estimations (see Supplementary Section 4) give the conversion efficiencies of 6 × 10^−8^ W^−1^ and 7 × 10^−9^ W^−1^ for the x- and y-polarized incidence, respectively. The marked difference in SHG conversion efficiency between the two polarizations highlights the strong influence of the MD mode, further confirming its critical contribution to the nonlinear response.

### SHG wavelength dependence

**Fig. 3:**
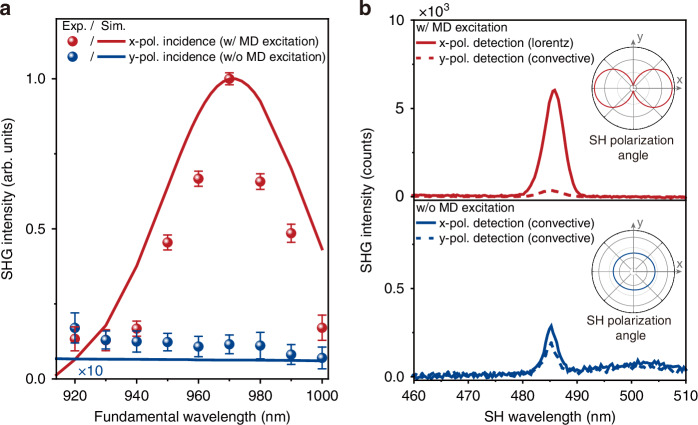
Nonlinear optical spectroscopy measurements and numerical simulations based on HDM. **a** SHG output from a single plasmonic nanocavity as a function of fundamental wavelength under x-polarized (red) and y-polarized (blue) incidence. Experimental measurements (dots) show excellent agreement with numerical calculations (solid lines) based on the hydrodynamic model. **b** SHG emission spectra at a fixed fundamental wavelength 970 nm, resolved by detection polarizations. The top panel shows the results under x-polarized incidence, where the MD mode is excited, revealing the dominant Lorentz contribution along x. The bottom panel corresponds to y-polarized incidence, where the MD mode is not excited, leading to a negligible Lorentz contribution and significantly reduced SHG intensity. The insets illustrate the simulated SH polarization angle for the two incidence configurations

Scanning the fundamental wavelength across the MD resonance, we observed strong variations in the SHG output. Figure 3a shows wavelength dependencies of the SHG output at the two orthogonal incident polarizations. When the incident polarization is parallel to the dimer axis (along the x-direction), the SHG signal in the vicinity of the MD resonance is prominently enhanced, reaching its maximum when the fundamental wavelength is 970 nm. In contrast, with y polarization, where the MD mode is not excited, the SHG intensity remains significantly lower across the entire covered wavelength range.

To understand the nature of the MD resonant SHG enhancement, we performed the hydrodynamic analysis of the SHG output. The calculated SHG dependencies on the fundamental wavelength are shown as solid lines in Fig. 3a. Without using any free-fitting parameters, we obtained an excellent qualitative agreement between the simulated and experimental SHG output. Specifically, near *λ*_MD_, the SHG intensity is prominently enhanced in the case of the x-polarized incidence (red). Moreover, the experimental redshift of the SHG maximum is clearly reproduced in the numerical simulations. Employing y-polarized fundamental radiation results in a weak, non-resonant SHG response in the entire simulated wavelength range (blue). In the simulations, we observe an enhancement by two orders of magnitude between the SHG outputs for the x- and y-polarized incidence (where the blue solid line has been magnified tenfold for better visualization). This sharp contrast between two polarized excitations is mainly attributed to the excitation of the MD.

### SHG polarization dependence

Taking advantage of the distinct symmetry of various SHG contributions, further insights into the origins of the resonant SHG output can be obtained from polarization-resolved studies. To that end, we set the fundamental wavelength to 970 nm (maximum SHG output under MD excitation) and installed a polarizer into the collection beam path. It enables selective control of the polarization of the registered SHG output, aligning it either along the x axis (x-pol. detection) or the *y* axis (y-pol. detection).

Under MD resonance excitation, the SHG output along the x- and y-directions differs by more than one order of magnitude. The SHG signal along the x-direction is predominantly driven by the Lorentz contribution, which is proportional to the cross-product of the electric and magnetic fields (see Supplementary Section 3.4). Whereas the SHG output along the y-direction originates primarily from the convective term. This behavior is illustrated in the top panel of Fig. 3b, highlighting the Lorentz-dominated resonantly enhanced SHG in our system.

In contrast, the bottom panel of Fig. 3b corresponds to the case where the MD mode is not excited. In this case, the Lorentz contribution becomes negligible, resulting in a significantly reduced SHG intensity. And there is no big difference between the x-pol. detection and the y-pol. detection, confirming that the main contribution comes from the vertical electric dipole^19^. This observation underscores the critical role of the MD mode in enhancing the nonlinear response.

Compared with previous studies on split-ring resonators^44^ and T-shaped structures^45^, here we have shown the design that facilitates Lorentz-dominated resonantly enhanced SHG through: (1) enhancing the magnetic field by exciting the MD mode; (2) improving the spatial overlap between the electric and magnetic fields. Most importantly, our dimer-on-film geometry enables substantial confinement of mode volume in an ultra-compact nanocavity.

**Fig. 4:**
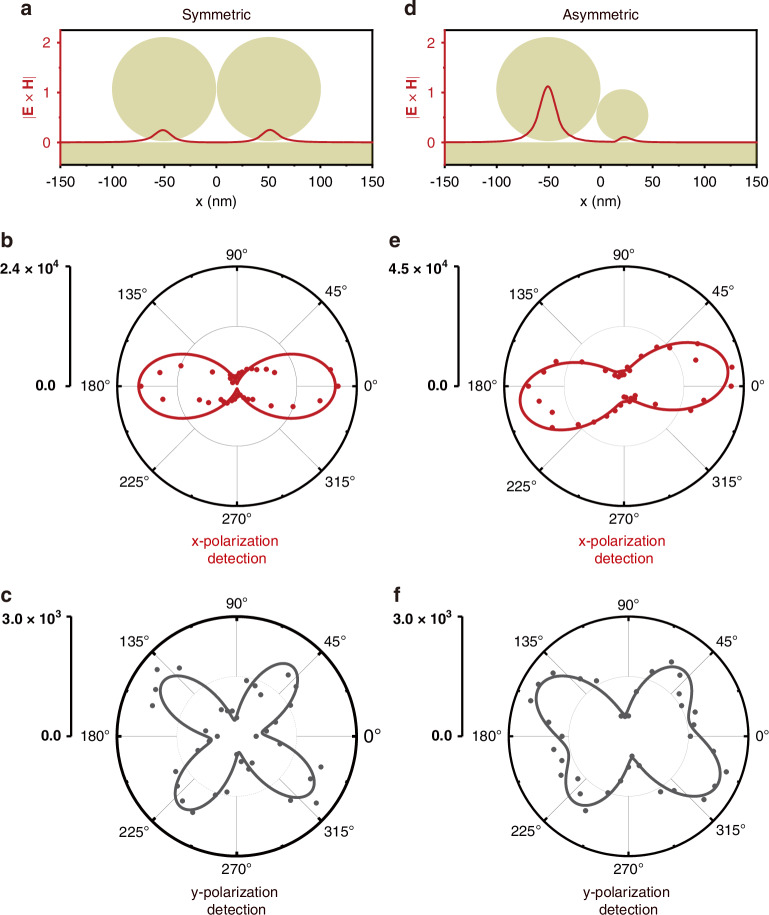
Polarization and symmetry analyses of SHG. Polarization-resolved SHG performances for dimer-on-film cavities. Schematic illustrations of **a** symmetric and **d** asymmetric dimer-on-film geometry, with the corresponding |**E** × **H**| distributions on the gold film surface. SHG intensities as a function of the incident polarization, collected along the *x* axis for the **b** symmetric and **e** asymmetric dimers. SHG intensities collected along the *y* axis for the **c** symmetric and **f** asymmetric dimers. The dots represent recorded SHG intensities, while the solid lines denote fitting results. The fundamental wavelength is set to 970 nm

Furthermore, by rotating the HWP in the incident path, we can separately obtain the dependencies of the x- and y-polarized SHG radiation on the fundamental wave polarization, as shown in Fig. 4. Under MD resonance excitation, the second-order nonlinear polarization at 2*ω* is dominated by the hydrodynamic Lorentz term, which is proportional to the cross-product of the electric and magnetic fields. Introducing an azimuthal angle α between the incident polarization direction and the dimer axis, the fundamental electric and magnetic fields can be expressed as: E_1_ ∝ (cos *α*, sin *α*, 1), H_1_ ∝ (0, cos *α*, 0). Here, we assume a negligible contribution from *x*- and *z*-directional magnetic fields, which are not enhanced by the MD resonance. The Lorentz contribution to the nonlinear polarization is thus ${{\boldsymbol{P}}}_{2\omega }^{{Lorentz}}\propto {{\bf{E}}}_{1}\times {{\bf{H}}}_{1}\propto (\cos \alpha ,0,{\cos }^{2}\alpha )$, whereas the Lorentz-driven SHG output takes the following form:

2 $${I}_{2\omega }^{\text{Lorentz}}={A}^{2}{\cos }^{2}\alpha +{B}^{2}{\cos }^{4}\alpha$$

where A and B refer to the amplitudes of the *x* and *z* SHG polarization components, respectively. The experimental polarization dependence can thus be fitted with Eq. (2) (Fig. 4b). The fitting results (|B/A | ^2^ ≈ 4) underscore the predominant orientation of the effective Lorentz-driven nonlinear polarization along the z direction.

When the detected SHG polarization is set parallel to the y-direction, the Lorentz contribution is inactive, and the SHG output is attributed to the Coulomb and convection terms in Eq. (1). Since both of these terms are phenomenologically related to electric quadrupole susceptibility, we describe the results with the ${\chi }_{{yxyx}}^{(2,Q)}$ quadrupole tensor component which for the SHG intensity gives ${I}_{2\omega }^{Q}\propto {\left|{{\boldsymbol{P}}}_{2\omega }^{Q}\right|}^{2}\propto {\left|{\rm{s}}{in}\alpha\, \mathrm{cos}\alpha \right|}^{2}$. Characterized by four distinct lobes, the experimental SHG intensity pattern is well reproduced by this dependence (Fig. 4c). The small isotropic background can be attributed to the electric dipolar ${\chi }_{z{zz}}^{(2)}$ contribution. Moreover, the intensity of the y-polarized SHG is one order of magnitude smaller than that of the x-polarized one, again highlighting the dominant role of the resonantly enhanced Lorentz contribution to the total SHG response.

### Symmetry breaking

To quantitatively assess the magnitude of the Lorentz-driven SHG enhanced by magnetic resonance, it is instructive to study it in systems where the symmetry breaking allows additional SHG contributions. Specifically, we introduce asymmetry by analyzing a dimer composed of the two nanoparticles with unequal diameters of 100 and 50 nm, as shown in Fig. 4d. Symmetry breaking is a well-established strategy for studying SHG^19,21^.

In the asymmetric case, the SHG response becomes more complicated, resulting in skewed polarization dependencies. In particular, symmetry breaking in the cavity has two effects on the SHG response. First, the electric dipole contribution ${{\boldsymbol{P}}}_{2\omega }^{{ED}}$, negligible in the symmetric case, now becomes prominent. Second, it results in a nearly threefold increase of the spatial overlap of the localized electric and magnetic fields (Fig. 4d). Both of these effects lead to an enhanced SHG response (note the scale in Fig. 4e). However, the polarization dependence of the x-polarized SHG still resembles the symmetric case, with a slight rotation of the two-lobe pattern. The striking contrast between the large difference in the diameters of the two nanospheres (×2) and the small (<15°) rotation angle is indicative of the dominant role of the two main SHG contributions (ED and MD) both of which are efficiently excited with the x-polarized fundamental radiation. Their interference with the dipolar ${\chi }_{z{zx}}^{(2)}$ and ${\chi }_{z{zy}}^{(2)}$ components enabled by lowering the dimer symmetry from C_2ν_ to C_1ν_ results in the rotation of the polarization angles, yielding the SHG maxima (see Supplementary Section 5).

The quadrupole SHG contribution becomes essential when the y-polarized SHG is registered (Fig. 4f). The interference of the ${\chi }_{{yxyx}}^{(2,Q)}$ and ${\chi }_{{yyxx}}^{(2,Q)}$ components results in a noticeable rotation of the retained four-lobe angular pattern compared to the symmetric dimer case. A joint fit of the two datasets indicates the necessity to consider a nonzero ${\chi }_{{yzy}}^{(2)}$ component and shows an enhanced quadrupole contribution to the nonlinear polarization (see Supplementary Section 5).

## Conclusions

In the ultra-compact dimer-on-film nanocavity, we have observed and analyzed the Lorentz force-induced magnetic SHG response. Both experimental and numerical results reveal the key role of the MD resonance in achieving a tenfold enhancement in the SHG output, along with its distinct polarization characteristics. The SHG conversion efficiency dominated by the magnetic mode is 6 × 10^−8^ W^−1^. The strong field confinement in this ultra-compact nanocavity improves the spatial overlap between the enhanced electric and magnetic fields, facilitating Lorentz-dominated resonantly enhanced SHG. Furthermore, by exploring symmetry breaking in the dimer, we enabled tunable contributions from electric and MDs to the SHG response. Through comprehensive polarization and symmetry analyses, we quantitatively describe the Lorentz-driven nonlinear behavior.

Our approach builds on foundational studies of nonlinear plasmonics^27^ and prior explorations of magnetic nonlinearities^44,45^, introducing a novel design framework by leveraging the Lorentz contribution to achieve a competitive SHG efficiency. Notably, this analysis of magnetic SHG enhancement differs from conventional nonlinear nanostructures that primarily focus on near-field electric enhancement^17^, thereby expanding the design framework for nonlinear-optical systems. This approach is general and can be readily extended to other plasmonic nanocavities, enhancing their versatility for nonlinear-optical systems. While bottom-up self-assembly approaches offer strong field confinement in the nanometer range, they introduce substantial polydispersity^58^, which may limit uniformity in large-scale production. For standard nanofabrication processes with high reproducibility, such as electron beam lithography^17^ and nanosphere lithography^59^ (NSL), fabrication tolerance remains a key challenge and should be carefully considered in experimental implementation. By leveraging an MD, our approach offers broader applicability, including tunability across a wider spectral range and hybridization with other optical modes. Significantly advancing our understanding of the nonlinear response of nanostructures, these discoveries hold promises for future innovations in nonlinear nanophotonics, facilitating the development of advanced nonlinear-optical systems at the nanoscale.

## Materials and methods

### Au dimer-on-film cavity preparation

A 2.2 mM sodium citrate solution (50 mL) in ultra-pure water was heated in a 100 mL three-necked round-bottomed flask under vigorous stirring for 15 min, with a condenser used to minimize solvent evaporation. Once boiling commenced, 0.3 mL of HAuCl_4_ (25 mM) was injected. Immediately after seed formation, the reaction was cooled to 90 °C, followed by the sequential addition of 0.3 mL sodium citrate (60 mM) and 1 mL HAuCl₄ (10 mM) with a 2 min interval. This process was repeated to control the particle size. A 1 mL Au nanoparticle solution and 200 μL of TEOS were then added to 30 mL of cyclohexane and reacted for 30 min to form a silica shell. The synthesized Au@SiO₂ nanoparticles were subsequently dispersed onto a ~ 200 nm thick gold film to form dimer-on-film nanocavities.

### Optical spectroscopy

Optical scattering spectroscopy at the single-particle level was carried out with a home-built polarization-resolved dark-field micro-spectroscopy system. The whole system was based on a commercial Confocal Raman Microscope (WITec Alpha300R) equipped with a ×100 dark-field objective (NA = 0.75). Using our home-built illumination arm as a light source, where the incident polarization can be continuously tuned, thus enabling both s- and p-polarized excitation. In addition, the incident angle θ can also be tuned within a finite range limited by the objective (see Fig. S2a).

SHG measurements at the single-particle level were performed using a home-built confocal microscope setup. The output of a Ti:Sapphire laser oscillator (Chameleon, 140 fs pulse duration, 80 MHz repetition rate) used as an excitation source was coupled to a dry objective lens with a high-numerical-aperture (MPlanApo ×100, Olympus, NA = 0.95). The SHG radiation was spectrally selected using a dichroic mirror and a short-pass filter (Thorlabs FESH0600) and recorded by a spectrometer (Andor SR500i) and a CCD camera (Newton). The polarization of fundamental radiation was controlled by a half-wave plate (see Fig. S2b).

### Full-wave simulations

Full-wave electromagnetic simulations were conducted using COMSOL Multiphysics V5.6, employing the finite element method to analyze plasmonic mode characteristics and nonlinear-optical responses. A perfectly matched layer was implemented to minimize spurious reflections at the computational domain boundaries. Mesh refinement was carefully performed, particularly in the gap regions, to ensure computational convergence. For nonlinear simulations, a perturbative approach was employed within the undepleted-pump approximation. This is justified by the fact that the SHG field intensity generated by the plasmonic nanocavity is orders of magnitude weaker than the fundamental field. Under this approximation, the SHG field does not couple back to the pump field, allowing the nonlinear process to be treated as two sequential linear scattering problems. First, a linear study at the fundamental frequency ω using the scattered field formulation allows us to analyze the MD and other mode characteristics of the nanocavity. Then, the associated nonlinear current distribution is calculated according to HDM (Supplementary Section 3).

## Supplementary information

Supplementary Information for Enhanced magnetic second-harmonic generation in an ultra-compact plasmonic nanocavity

## Data Availability

All data are available in the main text or the supplementary materials. Additional information can be obtained from the corresponding authors upon a reasonable request.
