# Supplementary material for: Enhanced magnetic second-harmonic generation in an ultra-compact plasmonic nanocavity
Source: Light Sci Appl. 2025 Sep 5;14:305. doi: 10.1038/s41377-025-01962-3 (PMC12411620; doi:10.1038/s41377-025-01962-3)
Supplement: Supplementary file 1 — Supplementary Information for Enhanced magnetic second-harmonic generation in an ultra-compact plasmonic nanocavity [file 41377_2025_1962_MOESM1_ESM.pdf]

Supplementary information

Enhanced magnetic second-harmonic generation in an ultra-compact plasmonic nanocavity

Yaorong Wang [1], Ilya Razdolski [1], Shixuan Zhao[1,2], Fan Yang[3], Xiu Liang[1,4], Yuri Kivshar[5], Dangyuan Lei[1,2]*

[1] Department of Materials Science and Engineering, Centre for Functional Photonics, and Hong Kong Branch of National Precious Metals Material Engineering Research Centre, City University of Hong Kong, Hong Kong S.A.R, 999077, China

[2] Department of Physics, City University of Hong Kong, Hong Kong S.A.R., 999077, China

[3] College of Physics and Key Laboratory of High Energy Density Physics and Technology of the Ministry of Education, Sichuan University, Chengdu, Sichuan 610065, China

[4] Advanced Materials Institute, Qilu University of Technology (Shandong Academy of Sciences), Jinan, Shandong, 250014, China

[5] Nonlinear Physics Center, Research School of Physics, Australian National University, Canberra, ACT 2601, Australia

[*] Correspondence: E-mail: dangylei@cityu.edu.hk

1 SAMPLE PREPARATION

Fig. S1(a) shows a schematic diagram of Au@SiO_2 nanoparticles, and the TEM image in Fig. S1(b) shows the approximate thickness of the silica shell. Gold film was prepared by depositing ~200 nm gold on a silicon wafer template through electron beam deposited at a rate of 0.5 ˚A/s (Kurt J. Lesker Company, PVD 75 Thin film deposition system). To obtain smooth gold film, silicon substrates are bonded to the freshly evaporated gold using a UV-curable glue. 3D Atomic force micrograph of the gold film showing a root mean square (RMS) surface roughness, of approximately 273 pm (see Fig. S1(c)). Under the wavelength and excitation power conditions used in our study, no detectable SHG signal was observed from the gold film, as shown in Fig. S1(d). Although the gold film itself does not exhibit significant SHG, it plays a crucial role in the cavity by breaking the inversion symmetry along the vertical direction and forming an essential part of the circular current required for the excitation of the MD.

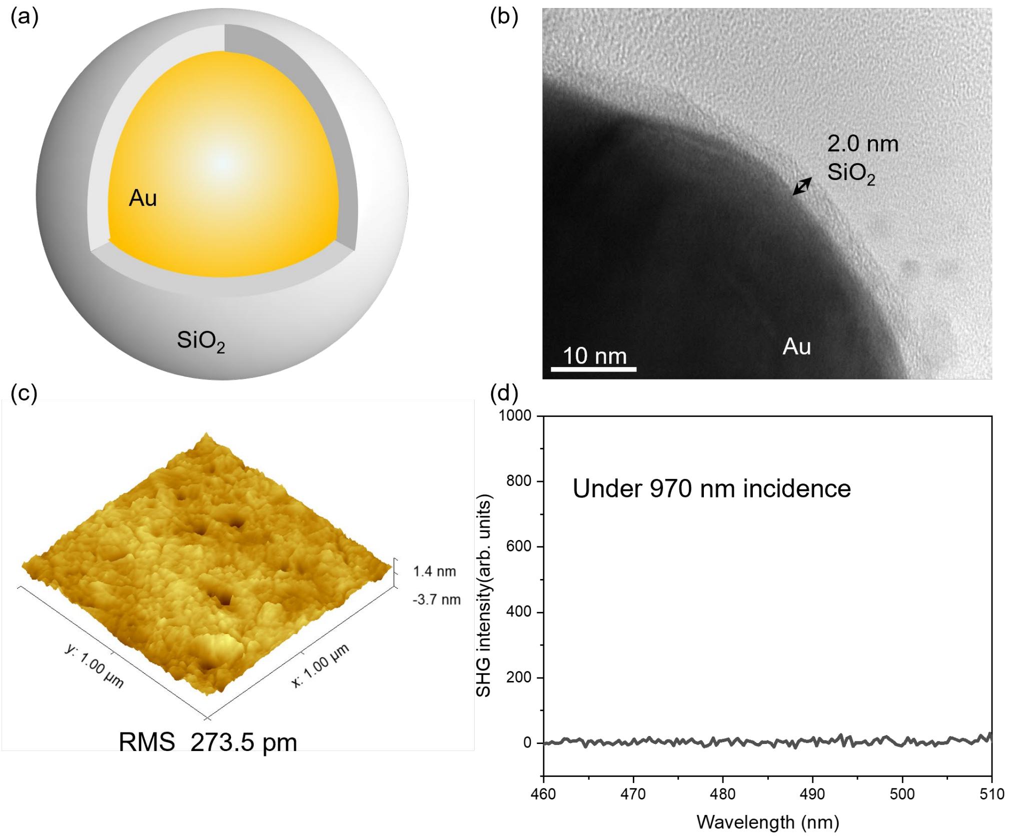

FIG. S1 (a) Schematic diagram and (b) TEM image of the Au@SiO2 nanoparticles. (c) 3D Atomic force micrograph of the gold film. (d) SHG response of gold film under 970 nm incidence.

2 OPTICAL SPECTROSCOPY

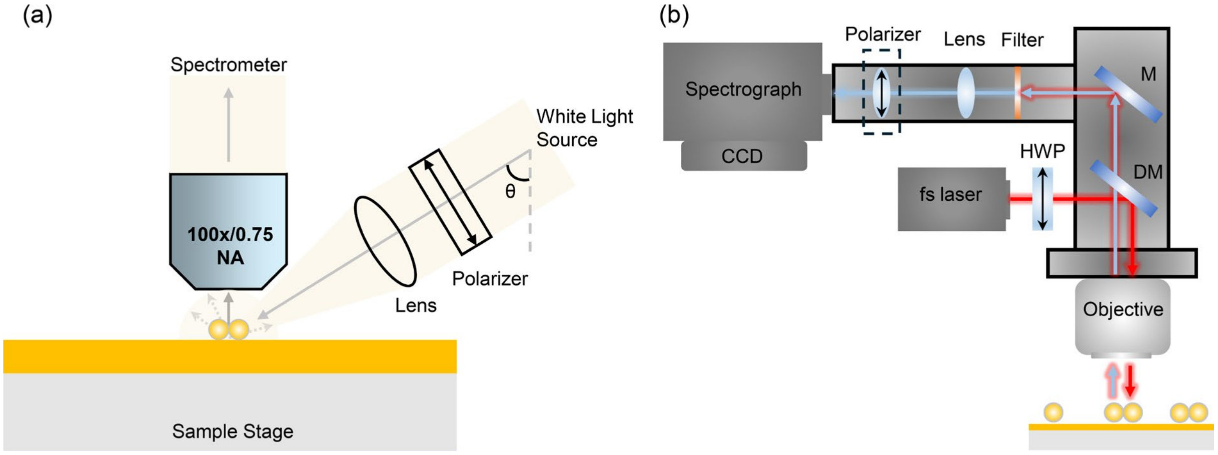

FIG. S2 Schematic diagram of (a) home-built polarization-resolved dark-field micro-spectroscopy system and (b) nonlinear experimental setup.

3 NUMERICAL SIMULATIONS

3.1 Dielectric function of gold

The dielectric function of gold in our simulations is modeled by incorporating both the free electron and bound electron contributions. The free electron response is described using the Drude model[1,2], given by:

$$\epsilon_{\mathrm{Drude}}(\omega) = \epsilon_{\infty} - \frac{\omega_p^2}{\omega^2 + i\gamma\omega} \tag{S1}$$

where ϵ_{∞} represents the high-frequency permittivity, ω_p is the plasma frequency, and γ denotes the damping rate. To account for the bound electron contributions at higher frequencies, we include additional Lorentzian terms in the dielectric function:

$$\epsilon_{\mathrm{total}}(\omega) = \epsilon_{\mathrm{Drude}}(\omega) + \sum_j \frac{f_j \omega_j^2}{\omega_j^2 - \omega^2 - i\gamma_j\omega} \tag{S2}$$

where f_j, ω_j, and γ_j are the oscillator strength, resonance frequency, and damping coefficient of the j-th bound electron transition, respectively. The parameters used in the Drude and Lorentzian terms were carefully optimized to match the experimental optical properties of gold, ensuring accurate representation of both interband transitions and free electron dynamics. This

combined model accurately captures the dispersive and absorptive properties of gold over a broad spectral range, ensuring reliable results for both linear and nonlinear optical simulations.

3.2 Calculations of the field distributions in the focal plane

The simulated scattering spectrum is shown in Fig. S3(a), corresponding to the SHG experimental conditions discussed in the main text. Note that a plane wave approximation is used here instead of using a tightly focused light beam in the simulation. Focusing of a normal incident Gaussian beam by a high-NA lens results in sizeable out-of-plane-polarized component of the near-field intensity. Based on the vectorial diffraction theory, the focal fields distribution of a linearly polarized beam can be calculated by [3]

$$\mathbf{E} = \begin{bmatrix} E_{\mathrm{x}} \\ E_{\mathrm{y}} \\ E_{\mathrm{z}} \end{bmatrix} = \begin{bmatrix} -i(I_0 + I_2\cos(2\varphi)) \\ -iI_2\sin(2\varphi) \\ -2I_1\cos(\varphi) \end{bmatrix} \quad \text{(S3)}$$

With

$$I_0(\rho) = \int_0^{\mathrm{k}\sin\alpha} F_0(\kappa)J_0(\kappa\rho)e^{iz\sqrt{\mathrm{k}^2-\kappa^2}}\mathrm{d}\kappa \quad \text{(S4)}$$

$$I_1(\rho) = \int_0^{\mathrm{k}\sin\alpha} F_1(\kappa)J_1(\kappa\rho)e^{iz\sqrt{\mathrm{k}^2-\kappa^2}}\mathrm{d}\kappa \quad \text{(S5)}$$

$$I_2(\rho) = \int_0^{\mathrm{k}\sin\alpha} F_2(\kappa)J_2(\kappa\rho)e^{iz\sqrt{\mathrm{k}^2-\kappa^2}}\mathrm{d}\kappa \quad \text{(S6)}$$

where $\kappa = \mathrm{k}\sin\theta$ is the spatial frequency, θ is the convergence angle between the rays in the focal image and the optical axis. The power contribution of the three field components in the focal plane can be approximately evaluated using the ratio:

$$\eta_i = \frac{\iint E_i^2\mathrm{d}s}{\iint (E_x^2+E_y^2+E_z^2)\mathrm{d}s}, i = x, y, z \quad \text{(S7)}$$

In the nonlinear experiment, the objective has a numerical aperture of NA = 0.95. By adjusting the incident angle θ to 20°, the proportion of each component is similar to the experiment. However, in polarization-resolved dark-field scattering measurements using our home-built illumination arm, the incident angle θ was constrained by the lens configuration to a range from 60° to 80°. The scattering spectrum presented in Fig. 1b of the main text was

obtained at an incidence angle of 70°. Notably, while variations in θ affect the scattering intensity, they do not shift the resonance peak position, as shown in Fig. S3(b). This observation confirms that the MD resonance frequency is independent of the incident angle. Such robustness arises from the localized nature of the MD mode, which is primarily determined by the intrinsic geometry of the nanocavity rather than the orientation of the incident wave.

3.3 Mode analysis

To better understand the properties of the MD, we present the enhanced magnetic field components in Fig. S3(c-e). The results clearly demonstrate that the magnetic field is predominantly oriented along the y-direction, consistent with the cavity's geometry and excitation conditions. Here, only the *xoz* plane is shown, while the y-direction remains the dominant component throughout the entire cavity. The alignment of the incident light can be achieved through polarization-resolved scattering spectra and image.

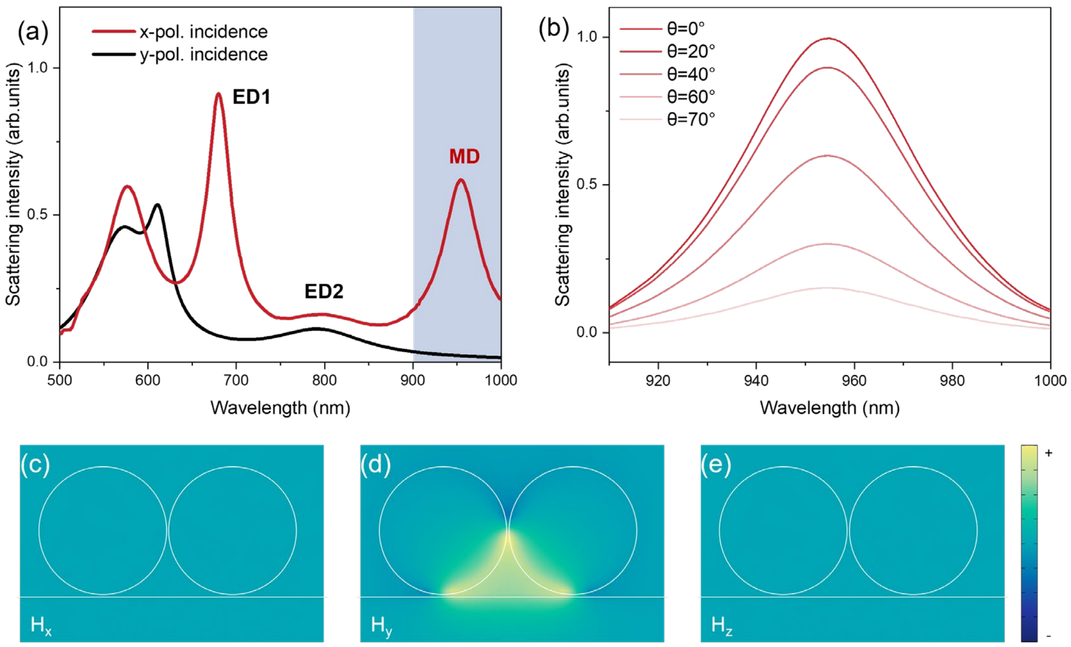

FIG. S3 (a) Simulated scattering spectra under two excitation polarizations. (c) Simulated scattering spectra of the dimer-on-film cavity under x-polarization excitation as the incidence angle θ increases from 0° to 70°. Spatial distributions of the enhanced magnetic field components within the nanocavity: (c) H_x, (d) H_y, and (e) H_z.

To get the difference between MD and other ED modes, we present distributions of the electric and magnetic fields in the ED1 and ED2 modes in Fig S4. For ED1, the maximum electric field amplitude enhancement factor exceeds 300 and the magnetic enhancement factor is 8. For the ED2 mode, the electric field amplitude enhancement reaches approximately 250, while the magnetic field enhancement is 3. It is seen that the magnetic field enhancement is not significant in these modes, and moreover, there is no substantial spatial overlap of the enhanced magnetic and electric field. Magnetic "hot spots" in these modes are not located in the triangular regions but in other gap regions where nearby currents form very localized and small loops.

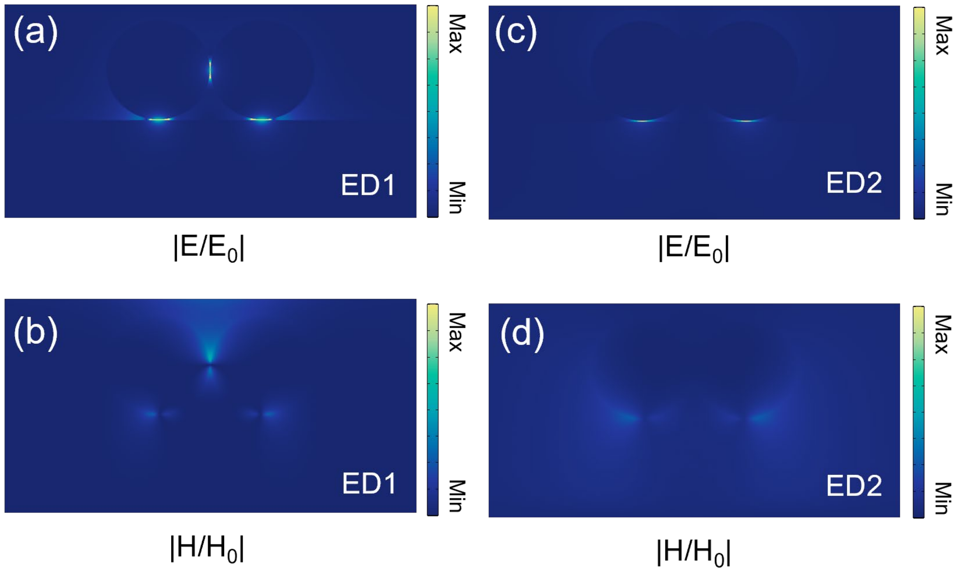

FIG. S4 (a) Electric and (b) magnetic fields at the ED1 resonance (at 660 nm). (c) Electric and (d) magnetic fields at the ED2 resonance (at 750 nm).

3.4 Hydrodynamic model

In the nonlinear optical simulations, we adopted the free-electron hydrodynamic model. Euler's momentum equation for the electron gas in a metal can be written as:

$$\frac{\partial \boldsymbol{v}}{\partial t} + \boldsymbol{v} \cdot \nabla \boldsymbol{v} + \gamma \boldsymbol{v} = \frac{e}{m_e^*}(\mathbf{E} + \boldsymbol{v} \times \mathbf{B}) - \beta^2 \frac{\nabla n}{n} \tag{S8}$$

where $\boldsymbol{v}$ and n are the electron velocity and density, $\mathbf{E}$ is the electric field, $\mathbf{B}$ is the magnetic flux density, m_e^* is the electron effective mass, e is the electron charge, and β is a constant originating in the Thomas-Fermi theory. The continuity equation is given by:

$$\nabla \cdot \boldsymbol{J} = -e\frac{\partial n}{\partial t} \tag{S9}$$

$$\boldsymbol{J} = \frac{\partial \boldsymbol{P}}{\partial t} = en\boldsymbol{v} \tag{S10}$$

where $\mathbf{P}$ is the free-electron polarization and $\boldsymbol{J}$ is the current density. Combining this with Maxwell's equations and expanding all the fields with harmonic time dependence ($e^{-i\omega t}$), gives the fundamental and second-order polarization vectors:

$$\boldsymbol{P}_1 = \frac{-1}{\omega+i\gamma}\frac{ne^2}{\omega m_e^*}\boldsymbol{E}_1 \tag{S11}$$

$$\boldsymbol{P}_2 = \frac{-1}{2\omega+i\gamma}\frac{ne^2}{2\omega m_e^*}\boldsymbol{E}_2 + \frac{1}{(2\omega+i\gamma)2\omega}\boldsymbol{S}_{NL} \tag{S12}$$

Equations S11 and S12 neglect nonlocal contributions arising from the electron pressure term, expressed as $\beta^2\,\nabla(\nabla\cdot\mathbf{P})$. This nonlocal term has been shown to play a significant role in shaping the optical properties of metallic systems with subnanometer gaps [4]. To assess the impact of nonlocality, we present in Fig. S5 a comparison between numerical results obtained with and without the inclusion of nonlocal contributions. The comparison reveals that, for the system considered in this study, the differences between the two approaches are minimal.

The nonlinear source S_{NL} for SHG can be written as:

$$\boldsymbol{S}_{\mathrm{NL}} = \frac{e}{m_e^*}\boldsymbol{E}_1(\nabla\cdot\boldsymbol{P}_1) + \frac{i\omega e}{m_e^*}\boldsymbol{P}_1\times\boldsymbol{B}_1 - \frac{\omega^2}{n_0 e}[(\nabla\cdot\boldsymbol{P}_1)\boldsymbol{P}_1 + (\boldsymbol{P}_1\cdot\nabla)\boldsymbol{P}_1] \tag{S13}$$

Equations S11 and S12 are solved in conjunction with Maxwell's equations, which, within the framework of harmonic propagation, are expressed as follows:

$$\nabla\times\nabla\times\mathbf{E}_1 - k_1^2\mathbf{E}_1 = \mu_0\omega^2\mathbf{P}_1 \tag{S14}$$

$$\nabla\times\nabla\times\mathbf{E}_2 - k_2^2\mathbf{E}_2 = \mu_0 4\omega^2\mathbf{P}_2 \tag{S15}$$

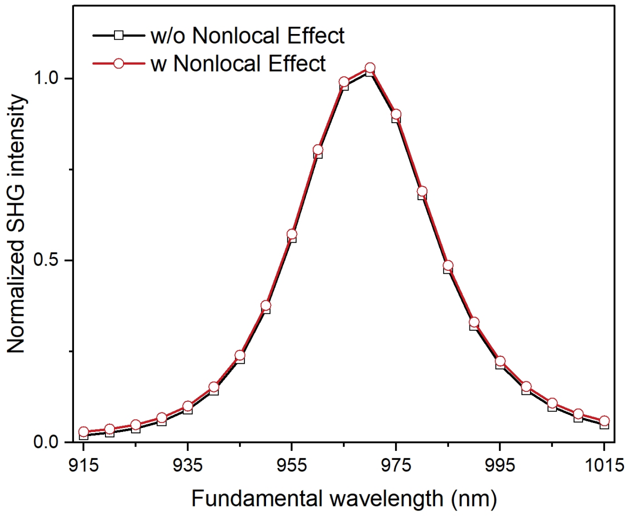

FIG. S5 Comparison of SHG intensity as a function of the fundamental wavelength with and without nonlocal contributions.

---

where $k_1 = \omega/c$ and $k_2 = 2\omega/c$, with c the light velocity in vacuum. Expressing these in terms of current density $\boldsymbol{J} = env$ gives:

$$\boldsymbol{J}_1 = \frac{i}{\omega+i\gamma}\frac{ne^2}{m_e^\star}\boldsymbol{E}_1 \tag{S16}$$

$$\boldsymbol{J}_2 = \frac{i}{2\omega+i\gamma}\frac{ne^2}{m_e^\star}\boldsymbol{E}_2 + \boldsymbol{J}_{NL} \tag{S17}$$

where:

$$\boldsymbol{J}_{NL} = \frac{ine^3}{m_e^{2\star}(2\omega+i\gamma)(\omega+i\gamma)}\left[\frac{1}{\omega}\boldsymbol{E}_1(\nabla\cdot\boldsymbol{E}_1) - i\mu\boldsymbol{H}_1\times\boldsymbol{E}_1 + \frac{1}{(\omega+i\gamma)}\big((\nabla\cdot\boldsymbol{E}_1)\boldsymbol{E}_1 + (\boldsymbol{E}_1\cdot\nabla)\boldsymbol{E}_1\big)\right] \tag{S18}$$

In the expression for the nonlinear current density $\boldsymbol{J}_{NL}$, the term containing $\boldsymbol{E}_1(\nabla\cdot\boldsymbol{E}_1)$ gives the Coulomb interaction, the term containing $\boldsymbol{H}_1\times\boldsymbol{E}_1$ gives the Lorentz magnetic force contribution, and the last two terms are the contributions from convection. Fig. S6 illustrates the contributions of each term in the hydrodynamic model to the SHG intensity, as well as the SH polarization angles of these terms under conditions with and without MD excitation. Fig. S7 shows the nonlinear far-field patterns of different contributions. The schematic diagram shows the direction of the cavity, and the far-field emission corresponds to

the electromagnetic analysis of Fig 1(f-h) in the main text. Fig. S8 shows the spatial distribution of the MD resonance second-order polarization components $P_x(2\omega)$ and $P_z(2\omega)$, respectively.

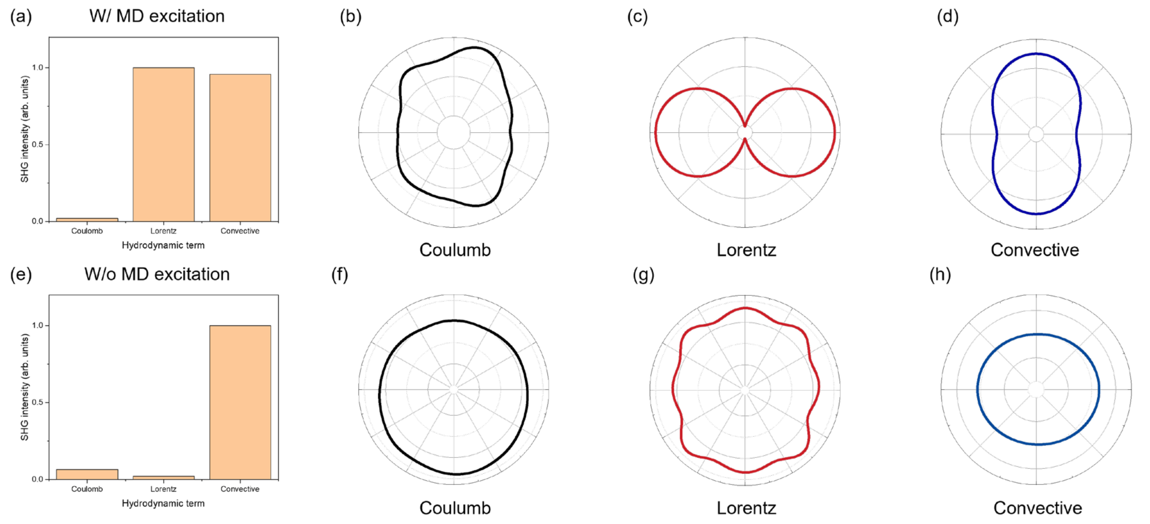

FIG. S6 (a) Normalized contributions of each term in the hydrodynamic model to the SHG intensity when MD is excited, the excitation wavelength is fitted to 970 nm. SH polarization angles of (b) Coulomb, (c) Lorentz, and (d) Convective term with MD excitation. (e) Contributions of each term when MD is not excited. SH polarization angles of (f) Coulomb, (g) Lorentz, and (h) Convective term without excitation of MD.

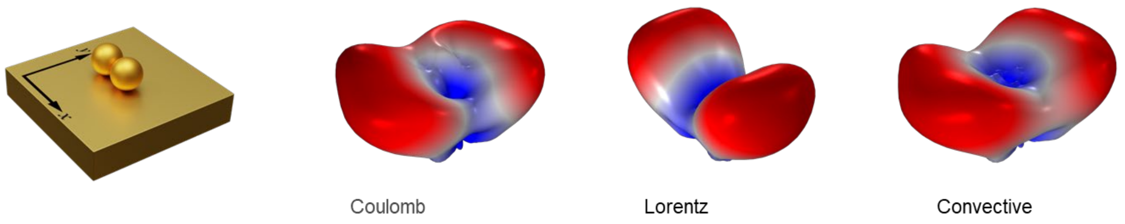

FIG. S7 (a) Simulated nonlinear far-field patterns of the different contribution for the dimer-on-film nanocavity. For calculations, $|E|^2$ is normalized to the full mode energy.

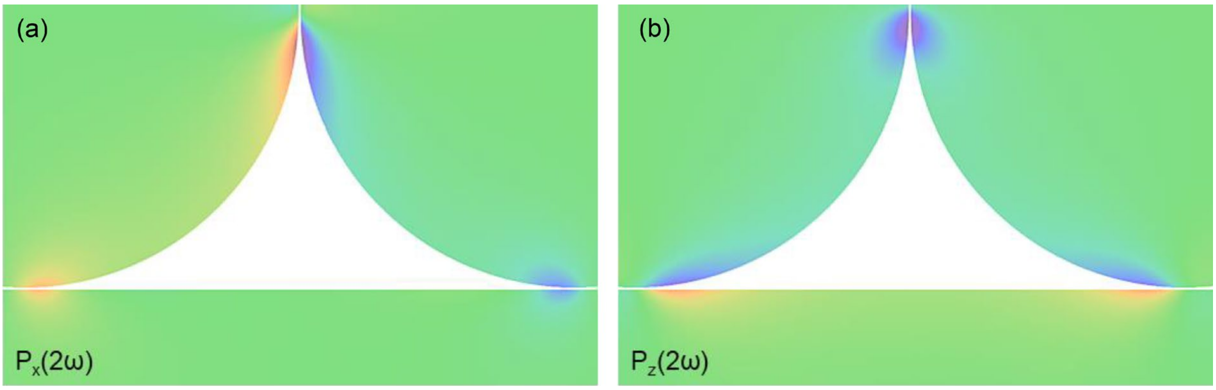

FIG. S8 Distribution of the second-order polarization, in the (a) x and (b) z directions, respectively, at the fundamental wavelength of 970 nm.

4 EVALUATION OF THE SECOND HARMONIC CONVERSION EFFICIENCY

To quantitatively evaluate the SHG efficiency of the cavity, we define the conversion efficiency as $\eta = W_2/W_1^2$. The W_1 is recorded using a power meter in front of the sample stage. Then the peak power can be obtained according to the repetition frequency and pulse width of the femtosecond laser. The W_2 can be experimentally evaluated by taking into account several parameters, i.e. the delivery efficiency of the objective lens, the transmission coefficients of all optical elements, and the quantum efficiency of the detector, respectively.

We selected a specific incident power (e.g., 53 W for the pulse peak power, see TABLE II), which, when focused by a 0.9 NA objective lens, produced a focal spot area of approximately 0.94 μm^2, corresponding to an intensity of around 10^9 W cm^{-2}. We also use another definition of SHG efficiency, which is direct ratio of the SHG power to the fundamental power: SHG efficiency = $P_{2\omega}/P_{\omega}$.

TABLE I. Collection efficiency.

Objective collection efficiency	Delivery efficiency	Detector Collection Efficiency
0.91	0.2	0.271

TABLE II. Analysis of the SHG efficiency.

Inciedence	SHG power (W)	Fundamental power (W)	SHG efficiency	$\eta_{SHG}(W^{-1})$
x-pol.	1.7×10^{-4}	53	3.1×10^{-6}	6.1×10^{-8}
y-pol.	1.9×10^{-5}	53	3.3×10^{-7}	6.8×10^{-9}

TABLE III. Comparison of the SHG efficiencies in different plasmonic nanostructures.

Year	Ref.	Material (Structure)	SHG efficiency
2015	5	Au (Nanoparticles onto Template-Stripped Substrates)	4.5×10^{-9} W^{-1}
2015	6	Au (V-and rod-shaped nanoantenna)	5×10^{-10} W^{-1}
2018	7	Ag nanocube on Au film	1×10^{-11} W^{-1}
2021	8	Au (particle-on-film)	3.6×10^{-7} W^{-1}
2023	9	Au (dimer antenna)	1.7×10^{-10} W^{-1}
2025	10	Ag (nanofinger-pair)	2×10^{-10} W^{-1}
2025	This work	Au (dimer-on-film)	6×10^{-8} W^{-1}

5 ASYMMETRIC DIMER ON FILM

Under further symmetry breaking of the dimer by changing the diameter of one of the spheres to 50 nm, the MD resonance wavelength shifts to shorter wavelengths. Approximately a two-fold enhancement of SHG is observed at the MD resonance compared to the symmetric dimer. This enhancement arises not only from the contribution of electric dipoles but also from the increased overlap of the cross product of the fields with the gold, as shown in Fig. S9.

Owing to a large number of non-zero second-order susceptibility components of both dipolar and quadrupolar origin, in the polarization dependence analysis we aimed at reducing the number of fit parameters as much as possible. This means that only absolutely essential $\chi^{(2)}$ components were considered in the fitting procedure while the others were omitted.

In the asymmetric case, the total SHG output $I_{2\omega}^{\mathrm{AS}}$ can be expressed as a sum of the MD-Lorentz $P_{2\omega}^{\mathrm{Lorentz}}$ and electric dipole $P_{2\omega}^{\mathrm{ED}}$ contributions.

$$I_{2\omega}^{\mathrm{AS}} \propto \left|P_{2\omega}^{\mathrm{Lorentz}} + P_{2\omega}^{\mathrm{ED}}\right| \quad \text{(S19)}$$

The experimental polarization dependence (red line in Fig. 4(e) in the main text) can thus be fitted with:

$$I_{2\omega,x}^{\mathrm{As}} = A^2\cos^2\alpha + B^2\cos^4\alpha + (D_1\cos\alpha + D_2\sin\alpha)^2 \quad \text{(S20)}$$

where D_1 and D_2 refer to the amplitudes of the dipolar *zzx* and *zzy* components, respectively. These components are non-zero in asymmetric dimers only.

For the y-polarized SHG, the experimental polarization dependence (black line in Fig. 4(f) in the main text) can be fitted with:

$$I_{2\omega,y}^{\mathrm{As}} = (C_1(\sin^2\alpha - \cos^2\alpha) + C_2\sin\alpha\cos\alpha)^2 + E^2\cos^2\alpha \quad \text{(S21)}$$

where C_1 and C_2 refer to the amplitudes of the quadrupole *yxyx* and *yyxx* components, respectively. E refers to the amplitude of the dipolar *yzy* component.

To better quantify the impact of the asymmetric dimer on the spatial overlap between the electric and magnetic fields, we have calculated the integral of $\mathbf{E} \times \mathbf{H}$ within the metal, using the *xoz* plane as an approximation. For the symmetric dimer, the integral yields a value of 8.2×10^{15}, while for the asymmetric dimer, it increases significantly to 2.28×10^{16}. This substantial increase demonstrates the enhanced spatial overlap in the asymmetric dimer, which plays a critical role in the Lorentz-driven SHG.

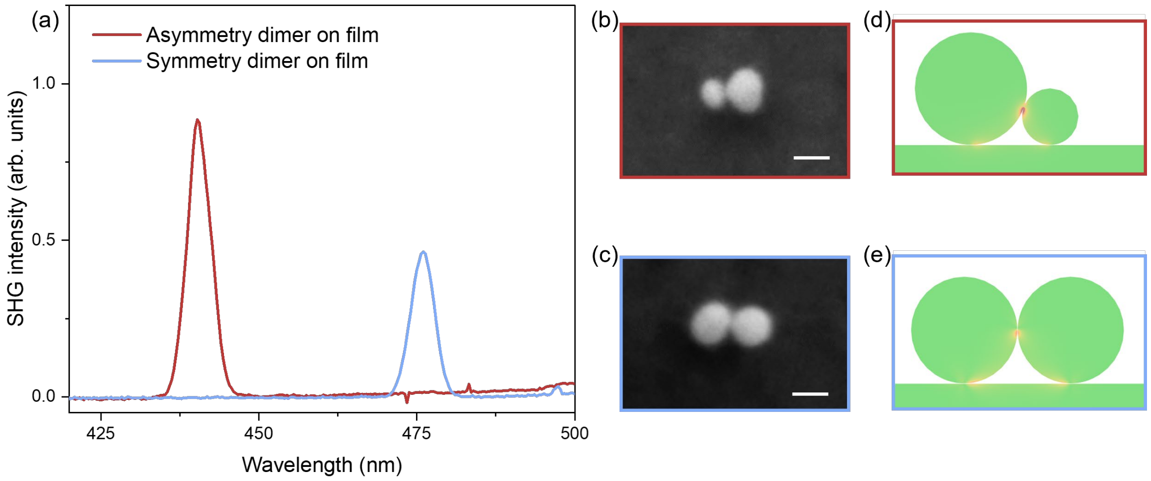

FIG. S9 (a) SHG emission spectra of an asymmetric (red) and symmetric (blue) dimer coupled to a gold film. Each spectrum was measured under the magnetic resonance excitation of the cavity. (b-e) SEM images and calculated Lorentz SHG mappings of (b, d) the asymmetric and (c, e) symmetric dimer on the gold film. The scale bar in the SEM images is 100 nm.

References

1. Johnson, P. B. & Christy, R. W. Optical constants of the noble metals. *Physical Review B* **6**, 4370–4379 (1972).

2. Olmon, R. L. *et al*. Optical dielectric function of gold. *Physical Review B* **86** (2012).

3. Bomzon Z, Gu M, Shamir J . Angular momentum and geometrical phases in tight-focused circularly polarized plane waves. *Appl Phys Lett* 2006; **89**: 241104.

4. Liu, D. et al. Probing the in-plane near-field enhancement limit in a plasmonic particle-on-film nanocavity with surface-enhanced Raman spectroscopy of graphene. *ACS Nano* **13**, 7644–7654 (2019).

5. Dong, Z. et al. Second-harmonic generation from sub-5 nm gaps by directed self-assembly of nanoparticles onto template-stripped gold substrates. *Nano Lett.* **15**, 5976–5981 (2015).

6. Celebrano, M. et al. Mode matching in multiresonant plasmonic nanoantennas for enhanced second harmonic generation. *Nat. Nanotechnol.* **10**, 412–417 (2015).

7. Zeng, Y., Qian, H., Rozin, M. J., Liu, Z. & Tao, A. R. Enhanced second harmonic generation in double-resonance colloidal metasurfaces. *Adv. Funct. Mater.* **28**, 1803019 (2018).

8. Li, G. C. et al. Light-induced symmetry breaking for enhancing second-harmonic generation from an ultrathin plasmonic nanocavity. *Nat. Commun.* 12, 4326 (2021).

9. Jessica, M. et al. Controlling field asymmetry in nanoscale gaps for second harmonic generation. *Adv. Opt. Mater.* **11**.21, 2300731 (2023).

10. Hu, J. et al. Second-harmonic radiation by on-chip integrable mirror-symmetric nanodimers with sub-nanometric plasmonic gap. *Nanophotonics* **14**.11, 1907-1915 (2025).
